# Precarious work, sickness absence and risk sharing between employers, employees and social insurance

**DOI:** 10.1093/eurpub/ckac129.376

**Published:** 2022-10-25

**Authors:** S Ose

**Affiliations:** SINTEF Digital, Health Research, SINTEF, Trondheim, Norway

## Abstract

**Background:**

The COVID-19 pandemic has revealed the importance of social protection systems including income security when health problems arise. Particularly the protection of those with precarious work felt short in some countries. For some time there has been an interest in the European variation in sick-pay schemes but still we still lack knowledge on country differences and similarities. This is particularly the case regarding precarious workers, while they have higher chances for sickness absence. Our aim is to understand, in the context of precarious work, the differences in risk sharing of sickness absence between employer, worker and social insurance.

**Methods:**

Data had been collected in a study on sickness absence follow-up regimes in nine countries (the Nordic countries (Sweden, Denmark, Finland, Norway and Iceland) and in Germany, the Netherlands, Belgium and the UK). Comparative statistics were collected and scholars familiar with their countries system were invited to answer a list of 51 questions on system characteristics. Data were re-analysed from the perspective of precarious work, using actor-network theory and insider-outsider theory of employment.

**Results:**

Countries with shorter employer periods of sick pay have stricter follow-up responsibility for employers as they are regarded gatekeeper except for The Netherlands. The tax-based systems that target all citizens offer more protection for precarious workers while the employee-focused systems define their target population more strictly, leaving precarious workers underserved. There is a large difference in how self-employed are supported or not.

**Conclusions:**

Despite small economic differences in the nine countries studied, the systems for dealing with sickness absence in the context of precarious work vary largely. Even though, in all systems those with secure jobs seem insiders and those with precarious work outsiders. Social protection systems should be updated to avoid an increasing inequality.

**Speakers/Panellists:**

**Anita Tisch**

Working time and Organization, Federal Institute for Occupational Safety and Health, Dortmund, Germany

**Angelique De Rijk**

Department of Social Medicine, Maastricht University, Maastricht, Netherlands

